# Implementation of informatics for integrating biology and the bedside (i2b2) platform as Docker containers

**DOI:** 10.1186/s12911-018-0646-2

**Published:** 2018-07-16

**Authors:** Kavishwar B. Wagholikar, Pralav Dessai, Javier Sanz, Michael E. Mendis, Douglas S. Bell, Shawn N. Murphy

**Affiliations:** 1 Massachusetts General Hospital, Boston, MA USA; 2 Harvard Medical School, Boston, MA USA; 3 University of California Los Angeles, Los Angeles, CA USA; 4 Partners Healthcare, Boston, MA USA

**Keywords:** I2B2, Docker, Containerization, Docker, High performance computing, Biomedical research, Information storage and retrieval, Medical records systems, Computerized, Software, Systems integration, User-computer Interface

## Abstract

**Background:**

Informatics for Integrating Biology and the Bedside (i2b2) is an open source clinical data analytics platform used at over 200 healthcare institutions for querying patient data. The i2b2 platform has several components with numerous dependencies and configuration parameters, which renders the task of installing or upgrading i2b2 a challenging one. Even with the availability of extensive documentation and tutorials, new users often require several weeks to correctly install a functional i2b2 platform. The goal of this work is to simplify the installation and upgrade process for i2b2. Specifically, we have containerized the core components of the platform, and evaluated the containers for ease of installation.

**Results:**

We developed three Docker container images: WildFly, database, and web, to encapsulate the three major deployment components of i2b2. These containers isolate the core functionalities of the i2b2 platform, and work in unison to provide its functionalities. Our evaluations indicate that i2b2 containers function successfully on the Linux platform. Our results demonstrate that the containerized components work out-of-the-box, with minimal configuration.

**Conclusions:**

Containerization offers the potential to package the i2b2 platform components into standalone executable packages that are agnostic to the underlying host operating system. By releasing i2b2 as a Docker container, we anticipate that users will be able to create a working i2b2 hive installation without the need to download, compile, and configure individual components that constitute the i2b2 cells, thus making this platform accessible to a greater number of institutions.

## Background

Informatics for Integrating Biology and the Bedside (i2b2), an open-source clinical data analytics platform, transforms patient data aggregated from the electronic health record (EHR) into a format optimized for various types and stages of research, including feasibility analysis, study design, eligibility criteria, cohort identification and recruitment, and population health studies [1, 2]. Conversely, I2b2 has the added functionality of allowing federated querying amongst participating i2b2 institutions, making it a central component in the informatics infrastructure for many national research institutions. Currently, over 200 institutions worldwide use i2b2 to query patient data.

I2b2, initially funded by the National Institutes of Health, has developed into an international project coordinated by the tranSMART Foundation, and has an active community of developers and researchers using and contributing to its development. I2b2 supports a sidecar approach wherein the platform aggregates a copy of patient data from the electronic health record (EHR) and provides query services in parallel to the EHR for research purposes. I2b2 software has been extended for importing C-CDAs and PCORnet clinical data models [3, 4], translation from HQMF [5] to FHIR [6–8], image management [9], federated querying, data analysis [10], and disease-specific analytics [11, 12].

The i2b2 platform has a modular architecture, which allows for its different components to be independently implemented and installed. In fact, an i2b2 installation, called a hive, consists of several i2b2 cells/services that provide different functionalities. Given the complexity of the i2b2 platform, creating a functional installation of the i2b2 platform can be challenging. Moreover, existing users find it difficult to apply patches for upgrading their installation. These difficulties pose a significant obstacle to i2b2 becoming available at a greater number of institutions. The goal of this work is to provide a simple method for the installation and upgrading of the i2b2 platform. Specifically, we hypothesized that containerization, which encapsulates the necessary components to run a program, can reduce the time required for i2b2 installation.

### Challenges for the installation and upgrade of I2b2

The i2b2 platform has a modular architecture, wherein the components (referred to as cells) communicate with each other using extensible markup language (XML)-based web services. This allows cells to be implemented and installed independently. The cells are categorized as “core” or optional: core cells are necessary for a functional installation, and optional cells add additional services, e.g. text processing capabilities. The platform is implemented using Enterprise Java, with a HTML-JavaScript User Interface. The source code is released as Open Source through GitHub. There is extensive web-based documentation for compiling and installing the i2b2 cells, and an online demonstration version of the software is available to showcase its functionality. However, despite the availability of online documentation, tutorials, and a community mailing list, new users require several weeks to create a functional i2b2 installation.

One challenge in installing i2b2 is the requirement of a moderate level of expertise in Enterprise Java and Java build tools for the compiling and deployment of the code. Another challenge is that installation steps must be adapted to newer versions of software dependencies that are released after the release of the i2b2 code and publication of i2b2 documentation. Finally, because i2b2 is designed to be flexible for installation on all popular operating systems (Linux, Windows, and macOS) and databases (PostgresSQL, Oracle, and Microsoft SQL Server), a wide combination of configurations are possible; therefore, following the exact steps to achieve a required specific configuration is difficult. The cumulative effect of these challenges poses a significant obstacle for the utilization of i2b2 by a greater number of institutions.

Once the i2b2 platform has been installed and populated with an institution’s data, it is essential to upgrade the installation at regular intervals. This involves replacing the i2b2 cells with newer code that adds new functionality or addresses security issues. Similarly, the database and operating system needs to be regularly patched. However, informatics teams often delay their efforts to upgrade the installation due to the risk of disrupting an operational i2b2 installation. One potential solution for these issues is containerization, which has recently been reported to be particularly useful for packaging scientific software [13–15]. Moreover, the use of Docker containers offers the potential to upgrade an i2b2 installation by replacing deployed container images with the latest images released into a central repository, such as Docker Hub.

### Containers facilitate packaging

Containerization is a type of operating system-level virtualization, where the operating system kernel allows the existence of multiple isolated processes that behave as separate individual computers, each with their own operating system. The containerization of software refers to the creation of a container image, which is a lightweight executable package that contains everything needed to run the software, including the executable code, runtime environments, and libraries. Containers run identically on any operating system that supports the container format. Containers encapsulate and isolate the software, thereby avoiding conflicts with other software running on the host machine.

Docker represents a containerization format that has become the de facto open standard due to its wide adoption in the industry. Containerization offers the potential to package i2b2 platform components into standalone executable packages that are agnostic to the underlying host operating system. The Docker format also offers the potential for users to install the entire i2b2 hive without the need to download, compile, and configure individual components that constitute the i2b2 cells. In this paper, we report on our efforts to create containers for the i2b2 platform in Docker format.

## Implementation

**Fig. 1:**
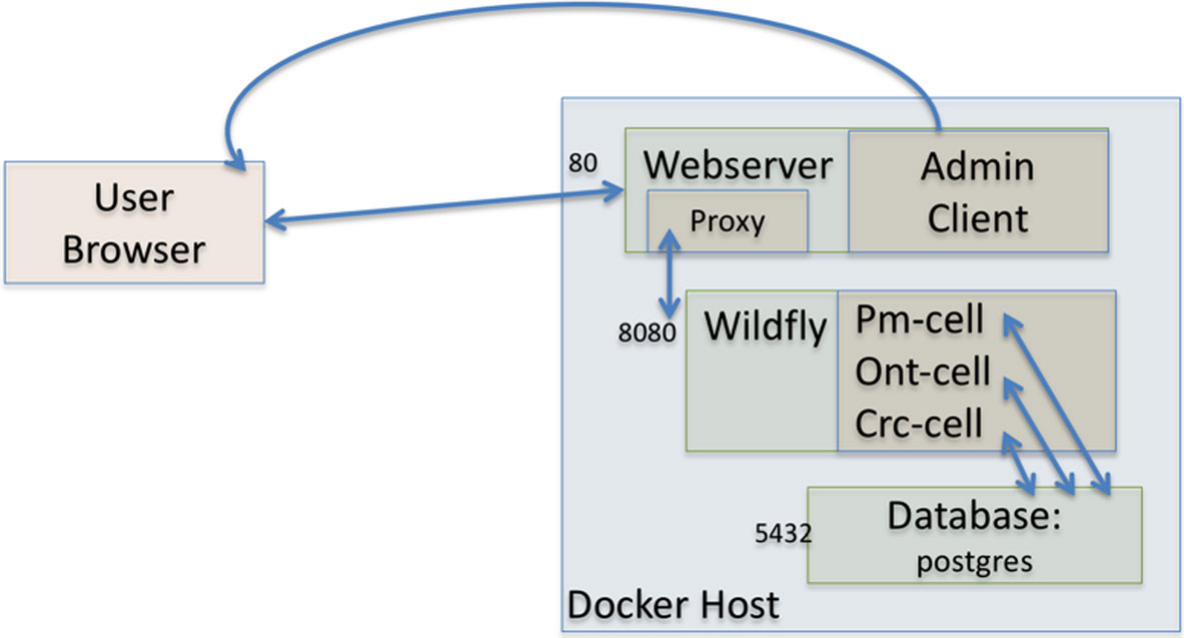
The architecture of an i2b2 Docker installation. The three major components of i2b2 — web server, application server, and database server – are encapsulated in three corresponding Docker containers. The containers are connected through a virtual Docker network

We created three Docker containers called ‘i2b2-web’, ‘i2b2-wildfly’, and ‘i2b2-pg’ to encapsulate the core functionalities of the i2b2 platform, as summarized in Table 1 and Fig. 1. The source code is published in GitHub (https://github.com/waghsk/i2b2-quickstart/) and the containers are available in Docker Hub.

**Table 1:** Comparison of the three Docker containers for i2b2

Name	Ports	Base image	Content	Function
I2b2-web (i2b2 code)	22, 443	Apache web server	Query web interface, HTML and JavaScript	User interface with the web pages and JavaScript AJAX calls trigger calls to i2b2 cells. This is the only component that is exposed to the external network.
I2b2-wildfly (web services or the cells)	8080, 9990	Jboss Wildfly	Core i2b2 cells: Data management (CRC), project management (PM), ontology management (metadata), Workspace	The cells listen for queries on port 8080 or 9990 and have a connection pool to the database.
I2b2-pg (Database)	5432	PostgreSQL	Database tables for installed cells.	The database only communicates with the i2b2 cells

Bash script to install i2b2 using the published i2b2-Docker containers

export IP=localhostdocker network create i2b2-netdocker run -d -p 5432:5432 --net i2b2-net --name i2b2-pg i2b2/i2b2-pg:p1docker run -d -e DS_IP='i2b2-pg' -p 8080:8080 -p 9990:9990 --net i2b2-net --name i2b2-wildfly i2b2/i2b2-wildfly:0.1docker run -d -p 443:443 -p 80:80 --net i2b2-net --name i2b2-web i2b2/i2b2-web:p1/run-httpd.sh $IPsleep 5;docker exec -it i2b2-pg bash -c "export PUBLIC_IP=$IP; sh update_pm_cell_data.sh;"

The i2b2-web image provides an Apache web server. It accepts a configuration parameter for the external internet protocol (IP address) [16]. At container boot-time, the external IP parameter is injected into the JavaScript for the user and administrative web client interface, and into the Apache webserver configuration.

The i2b2-wildfly image provides the JBoss WildFly server. The Apache Axis2 WAR archive is installed in the WildFly folder to enable web services. The source code for i2b2 cells is compiled into a WAR archive and installed in the WildFly server, along with XML configurationsto connect the data source to the WildFly server.

The i2b2-pg image provides the PostgreSQL Server. This includes a simulation dataset of 140 patients. This image accepts the external IP address and injects it into the database to reflect the URL for the i2b2 web services.

The three containers are secured in a user-defined Docker virtual network to enable their communication with each other. The server port of the i2b2-web image is exposed to the external interface, which allows users to connect to the i2b2 instance using a web browser. The configuration parameters used by the three containers are listed in Table 2.

**Table 2:** Configuration parameters for the i2b2 Docker containers

Name	Configuration parameters	Parameter description
I2b2-web (i2b2 client)	IP: external IP address	This parameter allows configuration of the Apache server to listen on an external interface, and the JavaScript, to connect the user interface to the webserver.
I2b2-wildfly (application server for i2b2 cells)	DS_IP: internet IP address for data-source DS_PORT: port for data source	These parameters allow configuration of the connection to the i2b2 database from the WildFly server (default is the i2b2-pg container running PostgreSQL
I2b2-pg (database)	IP: External IP address	This parameter is used to generate the URLs of the i2b2 web services, which are then stored in the database.

### Evaluation

For evaluating the functionality of the i2b2 Docker containers, we tested the deployment of the i2b2 containers on a local machine and on Amazon Web Services (AWS) Elastic Cloud Compute (EC2) servers, as described below:

1. Local On-premise Virtual Machine

We deployed a virtual machine, using VMWare Workstation Player, on a local computer with the following configuration: 4GB RAM, 10 GB HDD. We then installed Ubuntu 16.04 Operating System on it. We installed Docker Engine and its command line interface, and ran our scripts to download and start the i2b2 containers. We then executed our tests using atomated Python scripts to run queries against the i2b2 web services. The scripts emulate queries for particular concepts, and a valid response verifies the integrity of the i2b2 installation.

(2) Amazon EC2

We deployed an EC2 Server of the type “t2.medium” on Amazon AWS. We also enabled access to the web client server through a public IP. To test for successful installation, we tested if a user could successfully login using the i2b2 web client, then build and execute a query.

## Results

We were able to successfully install the i2b2 Docker containers on the local Ubuntu and Amazon Linux machines to create a demonstration installation of the i2b2 hive. On the Amazon machine, we found that the i2b2-Docker are installed and ready for use in 15 s. On local machines, we had to ensure that the operating systems supported Docker, and install the required Docker binaries. Once this was completed, we found the i2b2 Docker system took the same amount of time to install as on an AWS machine.

## Discussion

### Reproducible environments

Three containers were required to provide the functionalities of the i2b2 hive, as three independent processes are needed in order to run the platform: a web service, application, and the database servers. Docker runs each process in isolation within its container, which prevents conflicts with other installed programs in the hosting environment. As the containers themselves are initialized from the immutable base container images that we have created, the processes run in a system configuration that cannot change over time due to host system updates [17]. 

### Containers are faster and more explicit as compared to virtual machines

The i2b2 team has previously released virtual machines to provide a demonstration installation of i2b2. Although the virtual machines addressed the issue of packaging by capturing the entire software and development environment, they act as black boxes because they do not provide a recording of the steps needed to create the instance. However, Docker containers are distributed along with a Dockerfile, which provides a record of how the containers were generated. Consequently, Docker is better suited to ensuring transparency when compared to conventional virtual machines. Moreover, Docker images share the kernel with the underlying host machine, which enables significantly reduced image sizes and higher performance [18]. 

### Packaging and configuration and reproducibility of results

The i2b2 Docker containers offer an effective solution for packaging software components with the analytical software, along with the configuration settings. Docker has been recently reported to be useful for complex data retrieval and analysis workflows for Semantic web, workflow orchestration, [13] visualizing and analyzing gene networks [14], and phylogenomics [15]. The use of containers to distribute scientific software will help ensure the reproducibility of scientific results, [19, 20] and will facilitate the simultaneous publishing of data and code that can be repurposed for further research [21, 22]. Containerization in the i2b2 platform will facilitate reproducible performance of the i2b2 functionalities and plugin extensions.

### Containerization of database

The database container that we have provided for i2b2 is intended to be used with sample data, as containerized databases are known to have data loss risks, and are not currently recommended in production environments. After initial evaluation of the system, we recommend switching to a full-scale production database, and updating database configuration files in i2b2-wildfly Docker container to link it to the production database. Specifically, after the initial evaluation, the sample Postgres database container (I2b2-pg) should be stopped and the i2b2-wildFly container should be modified to point to a non-containerized production database.

### Limitations

We used PostgreSQL database in our study. However, several i2b2 sites are known to prefer other relational 2databases such as Oracle and Microsoft SQL. Our choice of PostgreSQL was due to the proprietary nature of the other databases that prohibit the sharing of containers in open-source. Nevertheless, our approach can be adapted to allow for connectivity to other databases, which represents a goal for our future efforts. Finally, the current study is limited to a demonstration dataset of 140 patients, and evaluation on larger, real-life datasets is necessary to ensure generalization of our results.

## Conclusion

Our study demonstrates that Docker containers can potentially reduce time and effort required to install i2b2 as compared to the conventional manual approach described in the i2b2 documentation. For institutions with preexisting i2b2 installations, the i2b2 Docker containers may simplify the technical hurdles of keeping their systems up-to-date, and allow for more efficient development of extensions. Similarly, for those considering adopting i2b2, the containers will serve to quickly create a proof of concept installation, which can be populated with the institutions data for use in a production environment. Overall, the i2b2 containers serve as a simplified i2b2 deployment system to enhance research infrastructure maintenance and development. We anticipate that by releasing i2b2 as a Docker container will improve platform accessibility to more institutions by enabling users to create a working i2b2 hive installation without the need to download, compile, and configure individual components constituting i2b2 cells.

## Availability and requirements

**Project name:** i2b2-quickstart.

**Project home page:** e.g. https://github.com/waghsk/i2b2-quickstart/

**Operating system(s):** Platform independent.

**Programming language:** Bash.

**Other requirements:** Docker.

**License:** i2b2.

**Any restrictions to use by non-academics:** none.

## Abbreviations

Amazon EC2: Amazon Elastic Cloud Compute
C-CDA: Clinical Continuity of Care documents
FHIR: Fast Health Interoperability Resources
HQMF: Health Quality Measures Format
i2b2: Informatics for Integrating Biology and the Bedside
PCORNet: Patient-Centered Outcomes Research Institute’s Network
